# Enhancing myogenic differentiation of pluripotent stem cells with small molecule inducers

**DOI:** 10.1186/2045-3701-3-40

**Published:** 2013-10-09

**Authors:** Jihong Chen, Qiao Li

**Affiliations:** 1 Department of Cellular and Molecular Medicine, Faculty of Medicine, University of Ottawa, Ottawa, Ontario, Canada; 2 Department of Pathology and Laboratory Medicine, Faculty of Medicine, University of Ottawa, Ottawa, Ontario, Canada

**Keywords:** Stem cells, Myogenic differentiation, Histone acetylation, Gene regulation, Nuclear receptor

## Abstract

Pluripotent stem cells are able to differentiate into many types of cell lineages in response to differentiation cues. However, a pure population of lineage-specific cells is desirable for any potential clinical application. Therefore, induction of the pluripotent stem cells with lineage-specific regulatory signals, or small molecule inducers, is a prerequisite for effectively directing lineage specification for cell-based therapeutics. In this article, we provide in-depth analysis of recent research findings on small molecule inducers of the skeletal muscle lineage. We also provide perspectives on how different signaling pathways and chromatin dynamics converge to direct the differentiation of skeletal myocytes.

## 

Pluripotent stem cells, regardless of their origin, can generate skeletal myocytes. However, the frequency of these cells to differentiate into skeletal myocytes is relatively low in the absence of inducing signals. Different types of mouse stem cells have been used as model systems to study the molecular mechanisms of myogenic differentiation. The commitment of these stem cells into skeletal muscle lineage recapitulates the cellular and molecular processes occurring in the early embryogenesis. However, the central issue is how to preferentially enhance the specification of muscle lineage for potential therapeutics. Therefore, understanding on a molecular level of how different cell signaling pathways and chromatin dynamics converge to regulate myogenic differentiation is imperative for identifying suitable small molecule inducers to efficiently generate skeletal myocytes. To this end, mouse pluripotent stem cells will continue to serve as valuable model systems because of their close resemblance to skeletal myogenesis *in vivo,* and their ease of manipulation in experimental procedures.

## Retinoid signaling in early development

In vertebrates, the proper distribution and metabolism of vitamin A is essential for normal embryonic development and growth [1]. Deficiency in vitamin A during early embryogenesis leads to congenital malformations and affects patterning and the development of many organ systems [2]. On the other hand, high concentrations of vitamin A, or pharmacological concentrations of retinoid acid (RA), the most potent natural form of vitamin A, have severe teratogenic consequences. These diversified effects of RA are mediated by multiple levels of effectors, including the enzymes that control RA metabolism, the cytoplasmic RA-binding proteins, and the RA receptors [3].

**Figure 1:**
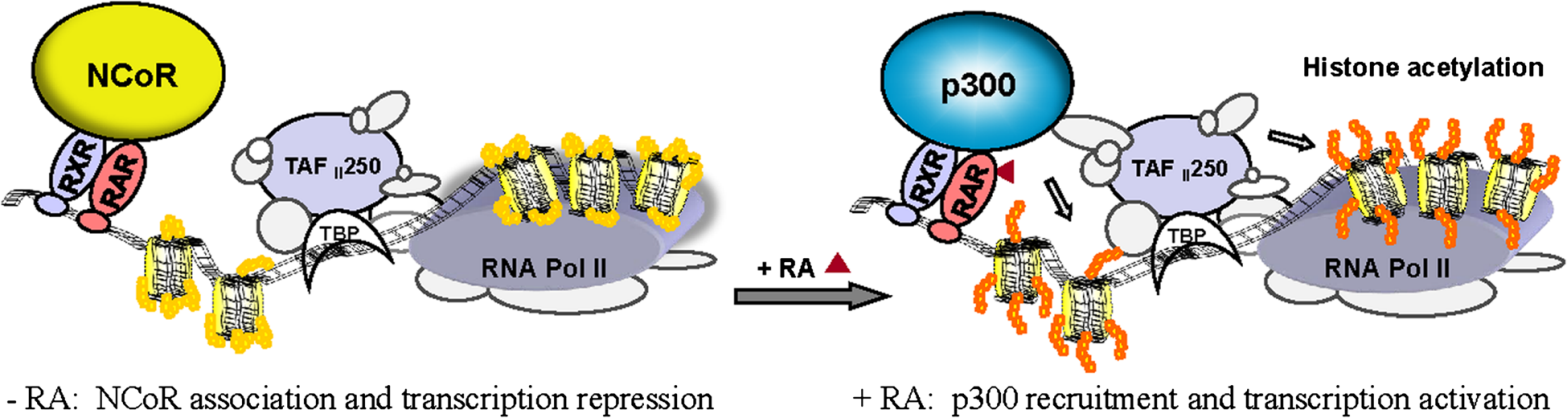
**Schematic diagrams of the bimodal function of the nuclear receptors.** In the absence of RA, the DNA-bound RAR-RXR heterodimer associates with the NCoR corepressor complex to repress gene transcription. Following RA induction, the RAR-RXR heterodimer recruits the p300 coactivator complex to initiate gene activation through chromatin remodeling and the release of RNA Pol II.

The retinoic acid receptors (RAR) are ligand-inducible transcription factors that regulate the RA-responsive genes in a bimodal mode. The functions of RAR depend on the retinoid X receptors (RXR). RAR binds to DNA constitutively with RXR as a heterodimer regardless of ligand binding. In the absence of a ligand, the DNA-bound RAR-RXR heterodimer functions as a transcription repressor by associating with the NCoR co-repressor complex. However, upon RA induction, it acts as an activator by recruiting the p300 coactivator complexes to activate gene transcription (Figure 1). As a result, NCoR is present at the RAR binding region in the absence of RA, whereas p300 is recruited to the region following RA signaling [4,5]. The RA-responsive promoters are often classified as pre-set, or poised promoters, because the TBP and Pol II complex associate to the TATA box constitutively [5].

In this bimodal model, the RAR-RXR dimer binds to the consensus DNA sequences, including the DR5 or DR2 motif, in which ligand induction is through the RAR, whereas RXR is generally considered a silent partner [6]. Nonetheless, in addition to RAR, the RXR is also able to dimerize with itself or with many other nuclear receptors to form permissive homodimers or heterodimers, in which the RXR is amenable to ligand activation.

Ligand-inducible nuclear receptors, including the glucocorticoid, thyroid, estrogen and androgen receptors, are a class of transcription factors that are capable of initiating dynamic chromatin changes in the promoter or enhancer regions by recruiting chromatin remodeling or modifying activities [7-13]. For example, the RA signaling is coupled with a global decrease in H3K27me3 modification, but an increase in histone acetylation, and is also coupled with the alterations in DNase I hypersensitive sites possibly through the dissociation of RAR-RXR dimer from SUZ12, and the recruitment of the histone acetyltransferase p300 [14-17].

There are three subtypes of RARs, namely RARα, RARβ and RARγ, which bind to all-trans and 9-cis RA [3]. The mice with each individual subtype of RAR knocked out are viable, appear to be normal, and have little developmental defects [18,19]. On the other hand, double RAR knockout mice exhibit a wide range of developmental abnormalities similar to vitamin A deficiency syndrome [20-23]. In fact, there is a large degree of functional redundancy between RARs which have important roles in many distinct stages of embryonic patterning and organogenesis [3].

The RXRs also consist of three subtypes, RXRα, RXRβ and RXRγ, which are activated by 9-cis RA [3]. The RXR knockout mice are well characterized as well. The RXRβ and RXRγ null mice are viable and seem to be normal [24,25]. However, RXRα null mutants die in utero and have myocardial and ocular malformations [24]. Most interestingly, the RXRα null mutants also exhibit developmental defects similar to fetal vitamin A deficiency syndrome [26,27]. Thus, RXRα is the main subtype involved in embryonic development.

Most interestingly, the compound RXR and RAR knockout mice recapitulate most of the developmental defects observed in the RAR double mutants [24,28], and RXRα-RAR is the major functional unit to mediate RA signaling during embryonic development [29]. Nonetheless, RXRs are also involved in many other signaling cascades and have the capacity to integrate multiple regulatory pathways as a ligand-bound receptor [30,31].

## Gene regulation and myogenic differentiation

Gene transcription is regulated by an integrated action of many *cis*-regulatory elements including the long-range enhancers, proximal regulatory elements, and promoters. Complex interactions among this assemblage of regulatory elements are vital to the control of target gene transcription [32]. In eukaryotic cells, genomic DNA, including these cis-regulatory elements, is organized with histones and further packaged into a higher order chromatin structure [33]. This chromatin organization establishes hierarchical platforms on both local and global levels for regulatory-protein interactions during epigenetic inheritance, cell fate determinations, and ultimately, the control of gene expression programs [34].

To decree the complex interaction of these cis-regulatory elements, the transcriptional coactivators or the HATs, recruited by sequence specific transcription factors play commanding roles in activating gene specific enhancers, and consequently, target gene transcription. For instance, active promoters are often associated with multiple histone modifications, whereas enhancers are associated with the HATs occupancy and histone acetylation [35-37]. Therefore, epigenetic and chromatin signatures have emerged as valuable marks to identify novel regulatory elements, in addition to DNA sequence motifs bound by potential transcription factors [38,39].

Skeletal myogenesis is a highly ordered process coordinated by multiple myogenic regulatory factors, such as Myf5, MyoD, myogenin, and Mrf4 [40]. While Myf5 and MyoD activate muscle-specific gene expression and commit the progenitor cells into skeletal muscle lineage, myogenin and Mrf4 largely regulate the late stage of myogenic differentiation, such as the fusion of myoblasts into myotubes [41]. Upstream of Myf5, the Wnt signaling and Shh from the dorsal neural tube and notochord act , respectively, as the positive regulators of Myf5 gene expression, whereas MyoD gene expression depends on Pax3 and Myf5 [42]. Additionally, genetic evidence in the mouse and ES cell model systems has established that the expression of Myf5 and MyoD genes depends exclusively on the HAT activity of p300 [43].

## Stem cells

Stem cells are excellent model systems for the studies of molecular mechanisms of cellular differentiation because of their abilities to differentiate into virtually all cell types *in vitro*. There are embryonic stem (ES) cells, adult stem (AS) cells, and induced pluripotent stem (iPS) cells, based on their derivative origins. The first evidence for the pluripotent nature of embryonic cells was obtained from studies of mouse embryonal carcinoma (EC) cells.

These EC cells, subcloned from teratocarcinomas, can be stably maintained as adherent cells and proliferate indefinitely in the tissue-culture dishes [44]. When cultured in the Petri dishes, they readily form cell aggregates which contain stem cells at the central part surrounded by epithelial cells. These cell aggregates, known as embryoid bodies (EBs), can develop extensive cavities and various cell types when subsequently grown as adhesive cultures [45]. For several decades, these EC cells have served as valuable model systems for the studies of early development and cellular differentiation, and paved the way for the isolation and establishment of mouse ES cells. Although, the pluripotent EC cells are much less used nowadays, they remain a useful model for the identification of small molecule inducers for myogenic differentiation [46].

## RA signaling and myogenic differentiation

One valuable model system for mechanistic studies of early development is the pluripotent P19 cell line. Isolated from an experimental teratocarcinoma, it exhibits a typical EC morphology and normal karyotype [47]. Like other EC cell lines, these P19 cells can grow in tissue-culture dishes as undifferentiated cells indefinitely, and differentiate into cell lineages of all three germ layers. More importantly, they are amenable for genetic manipulation to incorporate and express ectopic genes, and for selecting subclones and transfected stable clones which retain their ability to differentiate.

When grown in Petri dishes, the P19 cells readily form EBs. Mesoderm specification occurs at the early stage of EB formation, coinciding with an up-regulation of Brachyury T, a member of the T-box family of transcription factors [48]. However, EB formation *per se* does not lead to myogenic differentiation of the P19 stem cells, which requires additional inducing signals. When induced with small molecules, such as dimethyl sulfoxide (DMSO) or all-trans retinoic acid (RA), during EB formation, the P19 cells commit into the skeletal muscle lineage at a low frequency [49,50]. However, using combination of inducers, such as treating the EBs with both DMSO and RA, significantly increases the myogenic conversion of P19 stem cells [51].

The efficacies of P19 myogenic differentiation are influenced by the concentration of RA and the time line of treatments. Cells exposed to high RA concentrations (>10^-7^ M) develop into neurons and astrocytes, whereas EBs formed at the low concentrations (<10^-7^ M) differentiate into striated muscle [52]. The working concentration of RA for myogenic differentiation is typically around 5–30 nM, [46,53]. Nevertheless, the ability of P19 cells to generate skeletal myocytes is also influenced by other factors in the serum, and EB formation is a prerequisite for myogenic differentiation in these pluripotent cells [54].

Another valuable model system for mechanistic studies of myogenic differentiation is the mouse ES cells. They were first isolated in the early 1980s, from blastocysts grown on feeder-layer of division-incompetent mouse fibroblasts cells [55,56]. These ES cells express all markers of the EC cells, and can differentiate extensively *in vivo* and *in vitro*. The conditions for ES cell to differentiate *in vitro* are, in essence, the same as for the EC cells, depending on the process of EB formation [57]. However, the ES cells need to be maintained in inhibitory conditions to retain the undifferentiated state, because they are prone to spontaneous differentiation [58,59]. When grown in suspension culture without inhibitors, ES cells readily form EBs and consequently differentiate.

The early events of embryonic myogenesis are also closely recapitulated by EB differentiation of the ES cells into skeletal muscle lineage [60]. RA is also able to enhance the myogenic differentiation of ES cells. Specifically, RA affects the differentiation of ES cells into skeletal myocytes in a time- and concentration-dependent manner. Similar to the pluripotent P19 EC cells, high concentrations of RA (>10^-7^ M) induce neuronal differentiation of the ES cells, but suppress myogenic differentiation. Treatments of the EBs with low concentrations of RA (<10^-7^ M) at the stage of EB formation, enhance skeletal myogenesis, but inhibit cardiomyogenesis [61]. On the other hand, when low concentrations of RA are administered at the late stage of differentiation, skeletal myogenesis is inhibited, but cardiomyogenesis is enhanced [61].

Genetic manipulation has also been employed as an approach to induce myogenic differentiation of the ES cells. The premyogenic factor Pax3 plays an important role in embryonic muscle formation, acting upstream of muscle-specific gene program [41,62]. On the other hand, Pax7 is important for the maintenance of the muscle satellite cells [63-65]. Ectopic expression of Pax3 during EB differentiation enhances mesoderm formation and increases the myogenic potential of Pax3-induced ES cells [66]. Similarly, over-expression of Pax7 promotes the expansion of myogenic progenitors which possess muscle regeneration potentials [67]. Nonetheless, activating the myogenic signaling pathway with small molecular inducers, which can be easily administered into, or withdrawn from differentiation media, to direct myogenic specification remains a practical and appealing approach in view of potential cell-based therapies.

## Bexarotene as an efficient inducer of myogenic differentiation

RXR is critical for the early stages of embryonic development [24,26,27]. A recent study has identified bexarotene, a RXR-selective ligand, to be an effective inducer for enhancing myogenic differentiation in the pluripotent stem cells [46]. Interestingly, the RXR selective ligand enhances myogenic differentiation in a concentration-dependent manner. The range of bexarotene working concentration is wide, 10–1000 nM, which reflects the kinetics of ligand affinity for the receptor [46]. More importantly, high concentrations of bexarotene do not inhibit the differentiation of pluripotent stem cells into skeletal muscle lineage [46]. This is in stark contrast to the narrow concentration range of RA on myogenic differentiation *in vitro*[46].

During P19 myogenic differentiation, RA increases the expression of mesoderm factors Meox1 and Pax3 [53]. Although both Meox1 and Pax3 are important for myogenesis, over-expression of Meox1 *per se* is not sufficient to induce P19 myogenic differentiation [68,69]. Interestingly, bexarotene increases the transcript levels of Meox1 with a greater efficacy than RA, whereas RA has a larger impact on Pax3 gene expression than bexarotene [46]. Nonetheless, the temporal expression of muscle-specific gene program in bexarotene-enhanced P19 myogenic differentiation is similar to myogenesis *in vivo,* and the RXR ligand acts as an effective inducer for the specification of skeletal muscle lineage [46]. It is worth noting that bexarotene has efficacies comparable to RA at converting the P19 stem cells into muscle lineage [46]. While RA may enhance skeletal myogenesis by expanding the progenitor population [53], bexarotene appears to affect germ layer fate determinations, and more specifically, promote mesoderm differentiation [46].

ES cells respond to RA poorly with respect to myogenic differentiation, i.e., RA has a very low efficacy at converting the ES cells into skeletal muscle lineage [46]. DMSO is not suitable for ES cell differentiation due to its toxicity to the cells. However, bexarotene alone is able to specify the ES cells into muscle lineage at a relatively high efficacy [46]. Thus, bexarotene is a much more effective inducer than RA to enhance the differentiation of ES cells into skeletal muscle lineage [46]. In addition, bexarotene is much more effective at inducing the transcripts of mesoderm factor Meox1 than RA in the ES cells, but is less efficient at the augmentation of Pax3 transcripts [46].

**Figure 2:**
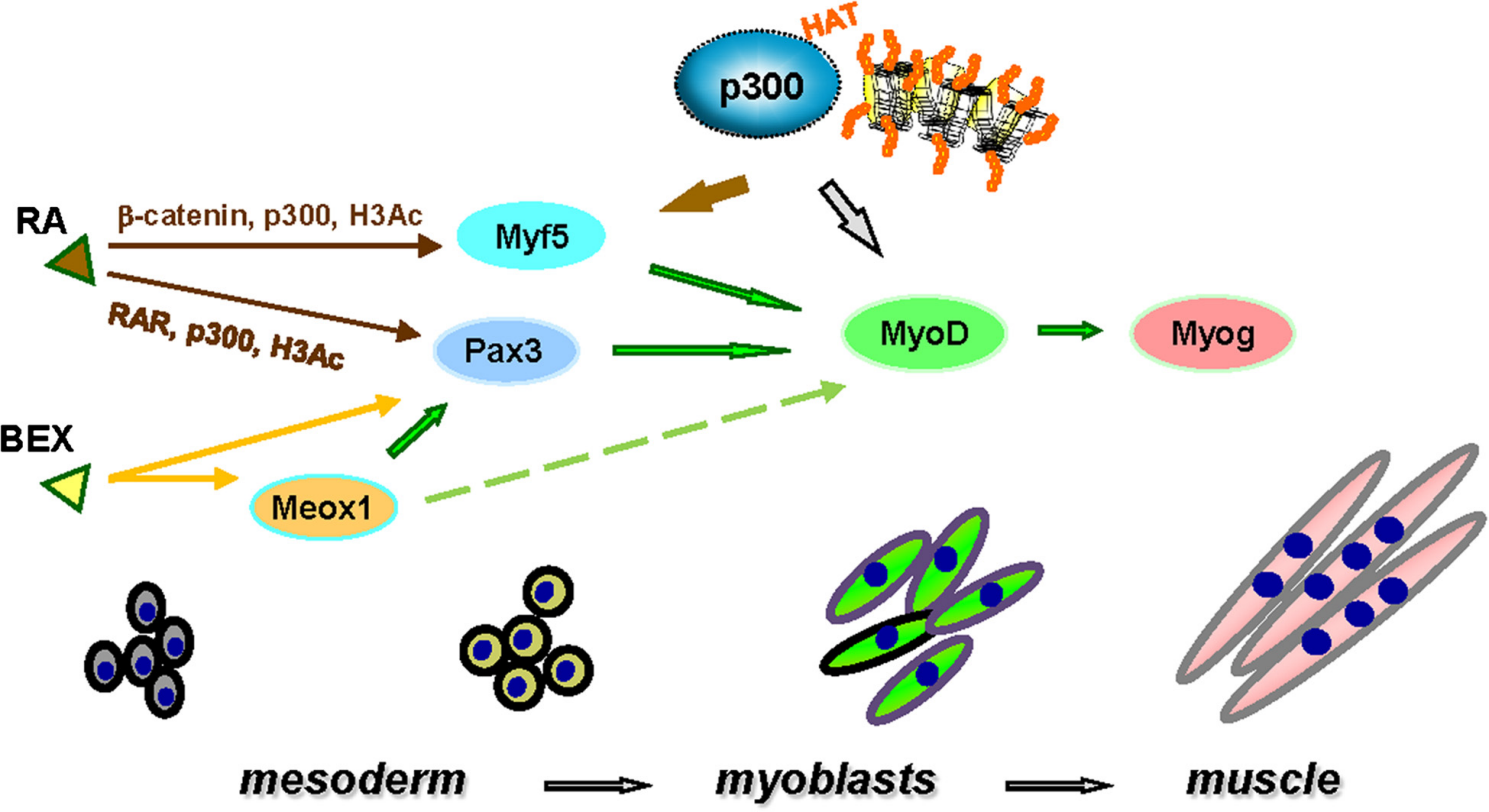
**Schematic presentation of the molecular mechanisms of myogenic differentiation.** RA directs myogenic differentiation through the regulation of Pax3 and Myf5 gene expression, whereas bexarotene (BEX) enhances the commitment of skeletal muscle lineage through the Meox1 and Pax3 pathways. The histone acetyltransferase p300 is directly involved in the regulation of myogenic differentiation through histone acetylation at the Pax3, Myf5, and MyoD gene loci. Solid arrows indicate identified regulatory pathways, whereas a dashed arrow suggests a potential regulatory pathway.

The finding of bexarotene to be a more efficient inducer than RA for myogenesis in the ES cell system is novel and significant [46]. In the ES cells, bexarotene alone is able to induce the expression of early differentiation marker Meox1, whereas RA depends on additional inducers to activate Meox1 expression (Figure 2). Thus, bexarotene may enhance the commitment of skeletal muscle lineage by fine-tuning the premyogenic transcriptional networks which favor the activation of the downstream myogenic program. Comprehensive systematic studies, such as RNA-seq analyses, will uncover additional early gene networks activated by RXR-specific signaling during mesoderm differentiation, identify novel early regulators of myogenic differentiation, and determine the molecular mechanisms by which the RXR agonist acts as an effective inducer of myogenic differentiation in the ES cells.

## Histone acetylation and myogenic enhancers

A long-range RAR binding site has been identified at the Pax3 locus [53]. Both RAR and RXR bind to this locus during the early stage of myogenic differentiation regardless of RA signaling, as determined by a real-time PCR based chromatin immunoprecipitation (ChIP) assay [46]. More importantly, the association of transcriptional coactivator p300 with this RXR-RAR binding site increased markedly following RA induction [46]. In addition, p300 is detected at a *Myf5* early enhancer at the early stage of myogenic differentiation by ChIP assay [70]. Thus, the *Myf5* early enhancer is also directly regulated by p300.

The association of p300 to the Pax3 locus depends on the ligand-bound RAR in an on-and-off mode, increased about 15-fold after RA induction [46]. In contrast, the occupancy of p300 at the *Myf5* early enhancer, which does not harbor a RAR binding site, increased only about 2-fold following RA signaling [70]. Interestingly, RA also increased the occupancy of β-catenin at the Myf5 enhancer by about 20-fold [70]. Nevertheless, histone acetylation increases at both the Pax3 locus and the Myf5 enhancer after RA induction (Figure 2). Therefore, RA regulates myogenic differentiation through p300-instigated histone acetylation in either DNA-bound RAR dependent or independent fashion.

In the differentiating myoblasts, the association of p300 to the MyoD enhancer is stepwise enriched at different regulatory regions, which positively correlates with increased histone acetylation in a discrete pattern [71]. Thus p300 is also directly involved in the early regulation of MyoD gene expression through specific histone acetylation (Figure 2). Nevertheless, the epigenetic marks for bexarotene-activated transcriptional networks or what transmit RXR specific signaling in myogenic differentiation remain to be determined. A comprehensive and systematic analysis by ChIP-seq will identify additional p300-dependent myogenic enhancers and uncover novel epigenetic marks to delineate the roles of p300 and histone acetylation in nuclear receptor-regulated stem cell differentiation.

## Cell-based therapeutics

Many diseases and conditions, including muscular dystrophy, aging, cancer, inflammation, starvation, AIDS, congestive heart failure and chronic obstructive pulmonary diseases, can cause muscle wasting disorders, which can be extremely debilitating and lead to serious physical disabilities. It would be difficult to use differentiated skeletal myocytes for tissue transplantation and muscle regeneration, due to the unique architecture of skeletal muscle tissue. Thus, muscle repair or regeneration may be best achieved through the enrichment or transplantation of the progenitor cells which are already committed to the muscle lineage but not yet fully differentiated into skeletal myocytes. However, many challenges remain regarding the efficacy of myogenic specification. Issues to note are what type of stem cell is the best source to generate the progenitor cells and what is the best strategy to enrich the desired progenitor cells for potential clinical application.

More importantly, the transplanted progenitors must supplement to both the muscle fibers and the muscle stem cell pool in a successful long term therapy for skeletal muscle regeneration or repair. Muscle satellite cells appear to be an idea cell source for muscle regeneration, because following transplantation, they not only generate muscle efficiently, but also replenish the satellite cell pool [72,73]. However, their therapeutic potential is restricted by their relatively low abundance in muscle. In addition, the *in vivo* regeneration capacity of these satellite cells is greatly reduced following in *vitro* expansion [74]. Finally, in the severe cases of muscular dystrophy, the regenerative source of satellite cells is often exhausted [75].

On the other hand, the ES cells can be unlimitedly expanded in tissue culture, while maintaining their potential for pluripotent differentiation. Moreover, ES-derived myogenic progenitors can be seeded in the muscle stem cell compartment [66,67]. Thus, ES cell-based muscle regeneration has some unique advantages. However, use of ES cells in muscle wasting disorders is curtailed by the low frequency of myogenic specification in the cultures and the difficulty of identifying and isolating the progenitor cells. The low frequency of ES cells to commit into skeletal muscle lineage is mostly due to the low efficiency of mesoderm formation during EB-differentiation in the absence of inducing signals.

To harness the potential of ES cells in muscle regeneration, we need to identify small molecule inducers that are capable of efficiently committing the ES cells into the skeletal muscle lineage. Attempts at using RA in ES cell cultures have yielded poor results, while the RXR ligand appears to be a better inducer for myogenic differentiation. However, the mechanisms involved have not yet been fully determined. A comprehensive knowledge of the differentiation cues in ES cultures and a better insight into the regulation of myogenic pathway *in vivo* will help us identify additional small molecule inducers and develop the optimal protocols to generate sufficient amount of myogenic progenitors for muscle regeneration or repair.

Small molecule inducers have been used to reprogram somatic cells, to maintain induced pluripotent states, and to directly control lineage specification. They also have the potential to control the endogenous cell populations for regeneration purposes. The advance of regenerative medicine will benefit tremendously from a deep understanding of chemical biology, and a better comprehension of the signaling pathways and the molecular mechanisms involved in cell fate determinations.

## Conclusions

Pluripotent stem cells possess a tremendous potential for the treatment of muscle-related diseases, because of their capacities to differentiate into the skeletal muscle lineage. However, small molecule inducers are required to direct the myogenic differentiation *in vitro* with an efficacy appropriate for viable cell-based therapies. Recent studies have uncovered the power of RXR-selective ligand to commit the ES cells into skeletal muscle lineage. Concerted systematic studies using stem cell differentiation as a model system will uncover novel early regulators and epigenetic marks important for myogenic differentiation*.* Pharmacological, or small molecule approaches to alter chromatin landscape for high efficiency of differentiation can then be identified. We will be able to develop non-toxic protocols with the optimal combination of inducers and conditions to commit the muscle lineage in view of generating muscle progenitors for clinical applications.

## Abbreviations

AS cells: Adult stem cells; ChIP: Chromatin immunoprecipitation; ES cells: Embryonic stem cells; iPS cells: Induced pluripotent stem cells; RAR: Retinoic acid receptor; RXR: Retinoid X receptor; RA: Retinoic acid.

## Competing interests

The authors declare that they have no competing interests.

## Authors’ contributions

JC and QL drafted the manuscript. Both authors read and approved the final manuscript.
